# Early Detection of Heart Failure with Autonomous AI-Based Model Using Chest Radiographs: A Multicenter Study

**DOI:** 10.3390/diagnostics14151635

**Published:** 2024-07-30

**Authors:** Emiliano Garza-Frias, Parisa Kaviani, Lina Karout, Roshan Fahimi, Seyedehelaheh Hosseini, Preetham Putha, Manoj Tadepalli, Sai Kiran, Charu Arora, Dennis Robert, Bernardo Bizzo, Keith J. Dreyer, Mannudeep K. Kalra, Subba R. Digumarthy

**Affiliations:** 1 Department of Radiology, Massachusetts General Hospital and Harvard Medical School, Boston, MA 02114, USApkaviani@mgh.harvard.edu (P.K.);; 2 Mass General Brigham Data Science Office, Boston, MA 02114, USA; 3 Qure AI, Mumbai 400063, Indiacharu.arora@qure.ai (C.A.);

**Keywords:** opportunistic screening, heart failure, artificial intelligence, chest radiograph

## Abstract

The opportunistic use of radiological examinations for disease detection can potentially enable timely management. We assessed if an index created by an AI software to quantify chest radiography (CXR) findings associated with heart failure (HF) could distinguish between patients who would develop HF or not within a year of the examination. Our multicenter retrospective study included patients who underwent CXR without an HF diagnosis. We included 1117 patients (age 67.6 ± 13 years; m:f 487:630) that underwent CXR. A total of 413 patients had the CXR image taken within one year of their HF diagnosis. The rest (n = 704) were patients without an HF diagnosis after the examination date. All CXR images were processed with the model (qXR-HF, Qure.AI) to obtain information on cardiac silhouette, pleural effusion, and the index. We calculated the accuracy, sensitivity, specificity, and area under the curve (AUC) of the index to distinguish patients who developed HF within a year of the CXR and those who did not. We report an AUC of 0.798 (95%CI 0.77–0.82), accuracy of 0.73, sensitivity of 0.81, and specificity of 0.68 for the overall AI performance. AI AUCs by lead time to diagnosis (<3 months: 0.85; 4–6 months: 0.82; 7–9 months: 0.75; 10–12 months: 0.71), accuracy (0.68–0.72), and specificity (0.68) remained stable. Our results support the ongoing investigation efforts for opportunistic screening in radiology.

## 1. Introduction

According to the Centers for Disease Control and Prevention (CDC), about 6.2 million adults suffer from heart failure (HF) in the United States [1]. In 2018, 379,800 death certificates mentioned HF. In 2012, HF had an estimated cost of USD 30.7 billion, encompassing healthcare services, medical treatments, and missed workdays. The HF cost is likely significantly greater now than in 2012 estimates [1]. Global statistics suggest that 64.3 million people worldwide suffered from HF in 2017, with an incidence rate of 1–20 HF cases per 1000 population, a 5-year mortality of 50–75%, and an annual healthcare cost of EUR 25,500 per year [2]. With increasing overall life expectancy and continued improvements in life-saving treatment options, the prevalence of HF is expected to rise [1,3]. The high prevalence of HF is likely related to its common risk factors, such as coronary artery disease, diabetes, hypertension, and obesity [3].

Timely diagnosis and initiation of treatment can slow the progression of HF and improve patient outcomes. Although HF can present with several symptoms, it is frequently first diagnosed in acute care settings of an emergency department or a hospital, where it carries a high mortality. A recent study suggested that among 959,438 patients with a new HF diagnosis, 38% of patients were diagnosed in acute care settings, and 46% of these patients had HF symptoms about 6 months prior to their diagnosis [4]. Underdiagnosis is more common in patients with lower socioeconomic status, black individuals, women, and those with comorbidities [4] Additionally, multiple studies have demonstrated a high prevalence of unknown or unrecognized heart failure and asymptomatic ventricular dysfunction in patients with known history of chronic degenerative diseases like diabetes, hypertension or COPD [5,6,7,8,9,10,11,12,13]. In such a context of delayed and overlooked HF diagnoses, we hypothesized that the most common imaging test of chest radiography (CXR) could be used as an objective and automatic tool for the opportunistic screening of HF. Therefore, we used a CXR-based AI tool to automate the quantification of cardiac silhouette size and pleural effusion to create an index for early HF detection. We performed a multicenter study to determine if the autonomous AI model can help in the early detection of HF using CXR signs.

## 2. Materials and Methods

### 2.1. Patients

**Figure 1:**
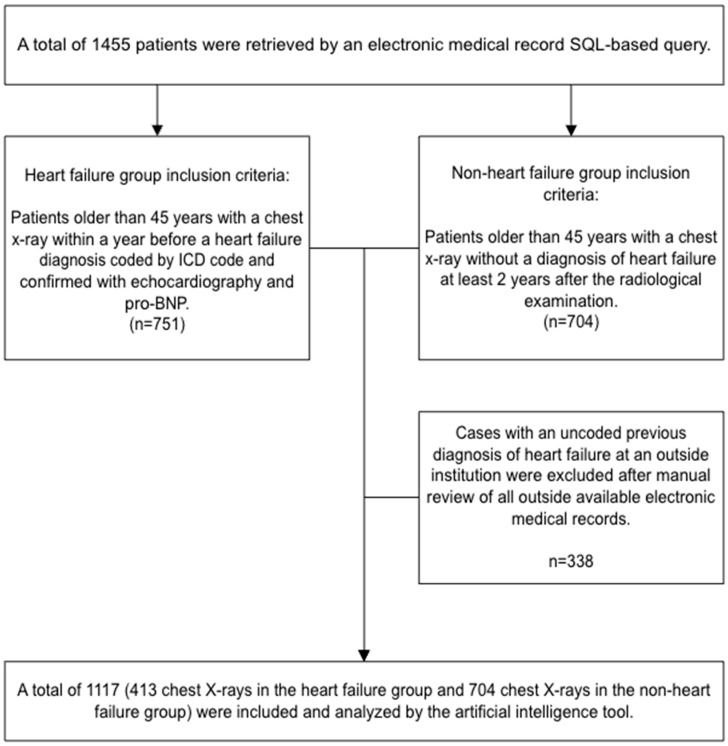
The flow diagram for inclusion and exclusion criteria.

The study included two groups of patients older than 45 years without a known HF diagnosis who underwent CXR. The inclusion criteria for the first group were as follows: baseline posterior–anterior projection CXR of patient without diagnosis of HF before or at the time of the examination; a minimum of one-year follow-up in the institutional medical records; and a clinical diagnosis of HF within a year of the CXR (by pro-B-type natriuretic peptide (NT-proBNP) levels and echocardiography at the time of diagnosis). The control group comprised >45-year-old patients without a known diagnosis of HF in their electronic medical records, either before or after the acquisition of their chest radiographs. Patients with the diagnosis of HF at the time of their CXR or before were excluded. Patients with only portable or anterior–posterior projection chest radiographs were excluded. There were 413 chest radiographs taken in the HF group and 704 chest radiographs taken in the non-HF group (Figure 1). 

All patients were identified using an SQL-based query on the Epic data warehouse (EDW, Epic, Verona, WI, USA) for querying diagnosis time, lab values, and imaging examinations in electronic medical records. All retrieved and eligible patients were included in the study in a consecutive manner without any exclusion. Two physician study coinvestigators (PKK, with three years of post-doctoral research experience, and EGF, with one year of post-doctoral research experience) manually reviewed the medical records of each patient to further confirm the SQL query results against the study eligibility criteria.

### 2.2. Chest Radiographs 

Once the two study groups, with and without the future diagnosis of HF, were confirmed, we exported and de-identified posterior–anterior projection chest radiographs from PACS (Visage 7, Visage Imaging Inc., San Diego, CA, USA). Chest radiographs with evaluation-limiting technical or quality issues (such as field of view not included, overlying anatomy, or oblique projection) were not included. Only one chest radiograph was included for each patient. 

Each radiograph was processed with a CXR-based AI algorithm (Qure.AI, Mumbai, India). The algorithm provided output on the probability of the presence of an enlarged cardiac silhouette and pleural effusion in addition to an index for heart failure risk based on the presence of enlarged heart size and pleural effusion. The heart failure risk index was calculated as a weighted sum of the probabilities of enlarged cardiac silhouette and pleural effusion. The algorithm exported the outputs into a comma-separated value (CSV, Microsoft EXCEL, Microsoft Inc., Redmond, WA, USA).

### 2.3. AI Algorithm

**Figure 2:**
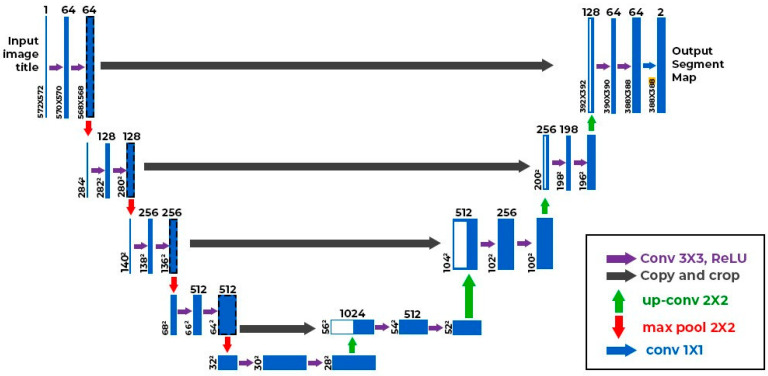
The UNET++ architecture for pleural effusion and enlarged cardiac silhouette algorithms (Qure.AI).

The enlarged cardiac silhouette algorithm (Qure.AI, India) is a UNET++-based segmentation model with an Efficientnet-v2 backbone (Figure 2). The model was trained to output segmentation masks of the heart and lungs to estimate the cardiothoracic ratio. The pleural effusion algorithm is a hybrid classification and segmentation model with the same UNET++ architecture, and outputs a probability score between 0 and 1 based on the probability of the presence of pleural effusion on the radiograph. It also outputs a segmentation map to mark the location of pleural effusions. 

The algorithms for both enlarged cardiac silhouette and pleural effusion are part of an AI software (qXR version 4.0) that can detect multiple other radiological findings in a frontal CXR image, in addition to enlarged cardiac silhouette and pleural effusion. The AI software device was trained with a large dataset of 4.2 million CXR images (about 70% from India, and the rest from the US, EU, Middle East, and South Asia) labeled by radiologists. The data were extensively filtered and cleaned to ensure quality, and then curated to minimize any racial or gender bias. Within the large training set, there were approximately 60,000 confirmed cases of pleural effusions and a similar number of cases with enlarged cardiac silhouettes. The device was internally tested using approximately 300,000 CXR images, with half the CXR images from similar sources and the other half from sites not included in the training process. 

### 2.4. Statistical Analysis 

Data were analyzed with the statistical software R Studio (V2023.06.1). We calculated the area under the curve (AUC) for receiver operating characteristic (ROC) analysis for the entire dataset (HF versus no-HF groups) and its optimal threshold. Next, we calculated stratified AUCs after dividing the chest radiographs into four subgroups according to the time between the CXR date and the date of HF diagnosis (subgroups—A: <3 months; B: 4 to 6 months; C: 7 to 9 months; D: 10 to 12 months) and three subgroups by left ventricle ejection fraction (<40% for low ejection fraction; 41–49 for mildly reduced ejection fraction; >50% for normal ejection fraction) reported in the cardiac ultrasound, when available. We used the optimal threshold obtained from the entire dataset analysis when calculating the AUC by time groups and ejection fraction groups. Mann–Whitney U and Kruskal–Wallis tests were performed to assess the statistical differences in the AI algorithm’s performance between the different subgroups. A *p*-value less than 0.05 was considered statistically significant.

## 3. Results

### 3.1. Overall Statistics

**Figure 3:**
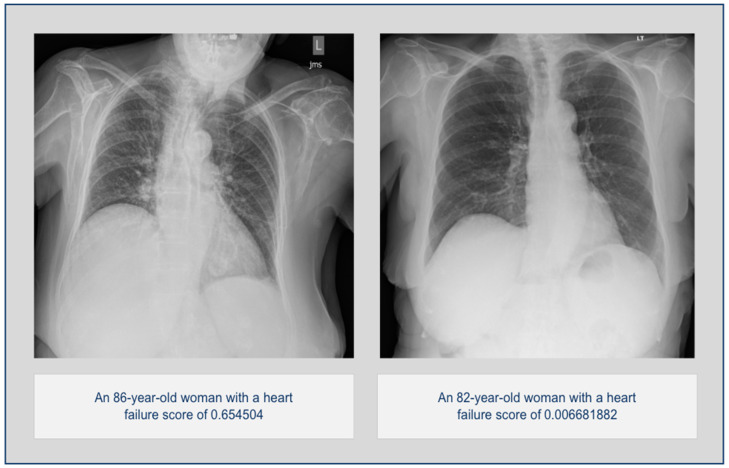
On the (**left**), a patient with a positive heart failure score was diagnosed with heart failure 10 months after CXR. On the (**right**) is a low-score patient without a diagnosis of heart failure.

The study included a total of 1117 chest radiographs from 1117 patients (female:male, 630:487; mean age, 67.60 years) from 11 hospitals. For the entire HF and non-HF groups, the algorithm had an AUC of 0.798 (95% confidence interval 0.772–0.824) using an optimal decision threshold of 0.389, with a sensitivity of 0.813, specificity of 0.684, and accuracy of 0.732. Two examples from each group are shown in Figure 3. 

The respective positive and the negative likelihood ratios were +1.888 and –0.471. There was no difference in model performance based on patient gender (female AUC = 0.807 vs. male AUC = 0.784) (*p* > 0.1).

There were significant differences in the three AI outputs (cardiac silhouette size, pleural effusion, and HF risk) between the HF and non-HF groups (*p* < 0.001). The average probability scores for enlarged cardiac silhouette and pleural effusions in the HF group were 0.537 ± 0.05 and 0.678 ± 0.29, and 0.487 ± 0.05 and 0.369 ± 0.32 for the non-HF group. The corresponding average HF risk scores between the two groups were 0.552 ± 0.19 and 0.292 ± 0.23, respectively.

### 3.2. Stratified Analysis

The AUCs for the subgroups based on the time intervals between CXR and HF decreased with increasing time intervals: <3 months—0.854; 3–6 months—0.821; 7–9 months—0.756; 10–12 months—0.717. The corresponding sensitivities exhibited a similar trend: 0.897, 0.851, 0.768, and 0.688 for the <3 months, 3–6 months, 7–9 months, and 10–12 months subgroups. The respective accuracies for these time subgroups were 0.717, 0.705, 0.695, and 0.685. The specificity for all groups was 0.684. We then used the heart failure probability scores in each group to perform a Kruskal–Wallis test. The differences in algorithm performance at four different time intervals were significantly different (*p* < 0.001). In the pairwise assessment, there was a strong significant difference (*p*-values < 0.003) between group A (<3 months) and groups C (7–9 months) and D (10–12 months), as well as between group B (3–6 months) and group D (10–12 months), but not between any of the two consecutive groups, such as groups A vs. B or groups C vs. D (*p* > 0.221). The contingency values for each group are depicted in Table 1.

The AUCs of the low ejection fraction, mildly reduced ejection fraction, and normal ejection fraction groups were as follows: 0.830, 0.804, and 0.795. The respective results for sensitivity were 0.857, 0.805, and 0.815, with a constant specificity for all groups of 0.684. The contingency table for this is depicted in Table 2. The respective accuracies across the three ejection fraction groups were 0.695, 0.690, and 0.720. There was no statistical difference in model performance across the three ejection fraction groups with HF (*p* = 0.061). 

A pairwise analysis demonstrated a significant difference (*p* < 0.019) between the low ejection fraction groups and the mildly reduced ejection fraction group, as well as between the low ejection fraction group and the normal ejection fraction group (*p* < 0.027). The difference between the normal ejection fraction and the high ejection fraction groups was not significant (*p* < 0.854).

## 4. Discussion

Our study shows that the HF risk index based on the probability of enlarged cardiac silhouette and pleural effusions in chest radiography can help detect the development of HF. Although we noted substantial predictive AUC and accuracy values for up to 12 months before the clinical diagnosis of HF, the AI performance was higher within 3 months of HF diagnosis than at 12 months from HF diagnosis. Our findings add to the growing body of evidence for opportunistic screening applications of AI algorithms for cardiovascular diagnosis from chest radiography.

The importance of early diagnosing heart failure and the exponential interest on the clinical application of artificial intelligence has encouraged the scientific community to study their potential clinical aplication. The processing of bigdata from patient demographics, signs and symptoms, laboratory results, and other clinical information from electronic medical records and wearables has been evaluated not only for diagnosing heart failure but also for predicting hospital readmissions and mortality in patient with HF [14,15,16,17,18]. Celik et al. reported on the use of an AI algorithm on 212 chest radiographs from a single healthcare practice to detect patients with undiagnosed HF. Their model assessed enlarged cardiac silhouette and pleural effusions with an index for HF diagnosis. The authors achieved an accuracy of 0.84, sensitivity of 0.90, specificity of 0.80, and a positive likelihood ratio of detecting HF in the chest radiographs of 4.54 [19]. The differences between the results in our study and Celik et al.’s investigation might be related to patient demographics, race, ethnicity, comorbidities, or underlying risk factors, as well as radiographic quality. Since enlarged cardiac silhouette and pleural effusions can be related to non-HF diagnosis, it is not surprising that our larger, multi-center study found low specificity for the AI algorithm. A much smaller study (n = 50 patients) with a Keras-based model (with all radiographs with enlarged cardiac silhouette labeled as positive for HF) and data from the National Institutes of Health (NIH) reported a higher accuracy of 0.82 [20].

Other AI algorithms using chest radiographs have targeted cardiopulmonary circulation, such as for the detection of high pulmonary arterial capillary pressure. Saito et al. reported an AUC of 0.86 for their AI algorithm for differentiating normal and elevated capillary pressures, compared with an AUC of 0.83 for the participating cardiologists (*p* = 0.24) [21]. Hirata et al. developed an AI model to differentiate between 115 patients with normal and elevated pulmonary capillary pressures with an AUC of 0.77 [22]. The authors explored several serum and radiological values, such as BNP, pulmonary arterial wedge pressure, and left ventricle ejection fraction to improve their AI performance for the detection of elevated pulmonary artery wedge pressure. Unfortunately, their study did not assess the model’s performance based on the classification of preserved or low ejection fractions. 

The use of chest radiographs has also been investigated for the classification of valvular diseases. Ueda et al. recently developed and validated an AI model that had an AUC from 0.83 to 0.92 to classify different valvular diseases, inferior vena cava dilatation (AUC of 0.85), and different groups of categories of left ventricular ejection fraction (AUC: 0.92) [23]. These results further support the research for disease detection in common radiological examinations.

We found that the low ejection fraction group had a significantly higher index than the mildly reduced and normal ejection fraction groups (AUC 0.830 vs. 0.804, and 0.795, respectively) (*p* < 0.01). This is biologically consistent with the pathophysiology of heart failure, as signs and symptoms are more frequent in the presence of noticeable structural changes in the low ejection fraction group. Since the heart can progresivelly adapt and pump excess volumes at the onset of cardiac insults due to the Frank–Starling mechanism, despite changes in cardiac size, some patients remain asymptomatic for a period of time. Over time, this mechanism becomes insufficient, and symptoms begin to appear [24,25]. Therefore, not every heart above normal size necessarily represents a case of heart failure, and other factors and results should be taken into account.

The major clinical implication of our study is the further reinforcement of the role of AI algorithms for detecting sub-clinical or undiagnosed HF with high AUC and accuracy. An early diagnosis of HF via chest radiography, the most frequently performed radiological test, can help initiate early treatment. The early treatment of HF can help improve the mortality and morbidity associated with HF, as reported in prior studies [26,27]. Our study also reinforces the need for radiologists to pay closer attention to the evaluation of cardiac silhouette on chest radiographs, particularly in posterior–anterior projections in the presence of concurrent pleural effusions. In the presence of such findings, referring physicians should consider triggering additonal investigations such as an echocardiogram or BNP to further study cardiac function in selected patients. [26,28,29,30]

Our study has several limitations. First, our study was retrospective and might not reflect the challenges of real-world clinical practice. Second, both enlarged cardiac silhouette and pleural effusions can exist without HF, a fact reflected by the low specificity noted in our study. Third, the AI algorithm did not specifically target the evaluation of pulmonary interstitial or airspace edema or the redistribution of pulmonary vasculature with pulmonary venous hypertension. Fourth, we did not specifically enrich the data with patients with non-HF causes of enlarged cardiac silhouette and/or pleural effusions. Fifth, our evaluation was limited to posterior–anterior projection radiographs only and did not include patients who underwent portable chest radiographs, which are extremely common in acutely ill patients and those visiting emergency departments or urgent care facilities. Sixth, we excluded patients with a diagnosis of HF at the time of their radiographs, which could have limited our ability to further stratify the results and gain a detailed understanding of the AI’s utility. Since our AI algorithm was built on a generic training dataset (without explicit annotation for radiographs with and without HF) for the detection of enlarged cardiac silhouette and pleural effusions, this could have resulted in lower-than-expected specificity.

## 5. Conclusions

We believe that future developments in the AI algorithm could target specific signs of pulmonary interstitial and airspace edema to improve performance, particularly from the point of view of specificity. Likewise, the AI-based evaluation of temporal changes in enlarged cardiac silhouette, pulmonary vasculature, and pleural effusions using serial radiographs can also help improve HF detection in underdiagnosed populations. Finally, a prospective study to assess the impact of the early radiographic prediction of HF will help determine if such an AI algorithm can lead to real financial gains and improvement in HF-related mortality and morbidity.

In conclusion, our autonomous AI model can help identify signs of HF up to 12 months prior to the clinical diagnosis of HF with a high and consistent AUC and accuracy, regardless of patient gender and imaging site. The detection of HF signs with an autonomous chest-radiography-based AI algorithm can help in the early diagnosis and treatment of unsuspected HF.

## Figures and Tables

**Table 1:** Contingency table of the four groups depending on the lead time between the radiological examination and the heart failure diagnosis.

<3 months group (n = 831; negatives:positives, 704:127)		Disease–Ground Truth	
		Positive	Negative
Model Inference	Positive	114	222
	Negative	13	482
4–6 months group (n = 805; negatives:positives, 704:101)		Disease–Ground Truth	
		Positive	Negative
Model Inference	Positive	86	222
	Negative	15	482
7–9 months group (n = 812; negatives:positives, 704:108)		Disease–Ground Truth	
		Positive	Negative
Model Inference	Positive	83	222
	Negative	25	482
10–12 months group (n = 781; negatives:positives, 704:77)		Disease–Ground Truth	
		Positive	Negative
Model Inference	Positive	53	222
	Negative	24	482

**Table 2:** Contingency table of the three ejection fraction groups.

Low ejection fraction group (n = 753; negatives:positives, 704:49)		Disease–Ground Truth	
		Positive	Negative
Model Inference	Positive	42	222
	Negative	7	482
Mildly reduced ejection fraction group (n = 740; negatives:positives, 704:36)		Disease–Ground Truth	
		Positive	Negative
Model Inference	Positive	29	222
	Negative	7	482
Normal ejection fraction group (n = 1019; negatives:positives, 704:315)		Disease–Ground Truth	
		Positive	Negative
Model Inference	Positive	257	222
	Negative	58	482

## Data Availability

The datasets presented in this article are not readily available due to restrictions from our Institutional Review Board.
